# Unbiased metagenomic next-generation sequencing of blood from hospitalized febrile children in Gabon

**DOI:** 10.1080/22221751.2020.1772015

**Published:** 2020-06-11

**Authors:** José Francisco Fernandes, Florian Laubscher, Jana Held, Isabella Eckerle, Mylène Docquier, Martin Peter Grobusch, Benjamin Mordmüller, Laurent Kaiser, Samuel Cordey

**Affiliations:** a Centre de Recherches Médicales de Lambaréné (CERMEL), Albert Schweitzer Hospital Lambaréné, Gabon; b Institut für Tropenmedizin, Eberhard Karls Universität Tübingen Tübingen, Germany; c German Center for Infection Research (DZIF) Tübingen, Germany; d Center of Tropical Medicine and Travel Medicine, Department of Infectious Diseases, Division of Internal Medicine, Amsterdam University Medical Centers, location AMC, University of Amsterdam Amsterdam, The Netherlands; e Division of Infectious Diseases and Laboratory of Virology, University of Geneva Hospitals Geneva, Switzerland; f University of Geneva Medical School Geneva, Switzerland; g Geneva Centre for Emerging Viral Diseases Geneva, Switzerland; h iGE3 Genomics Platform, University of Geneva Geneva, Switzerland; i Department of Genetics and Evolution, University of Geneva Geneva, Switzerland

Dear Editor,

Viruses represent the major cause of febrile consultations in children across sub-Saharan Africa [1,2], but considering their high number and the need of specific molecular tools targeting each of them individually, these agents are rarely characterized. This prevents drawing a clear picture of the causes of fever in this population and clinical guidelines are potentially poorly adapted to local epidemiology. Indeed, the majority of febrile cases are diagnosed as “of unknown origin”. Such clinical uncertainty is known to be an important driver of inappropriate antibiotic use [3] and makes it impossible to predict, detect or evaluate potential outbreaks or emerging infections. Further investigations of viral epidemiology in sentinel patient groups of Sub-Saharan Africa could help reduce uncertainty and aid the formulation of evidence-based guidelines for disease surveillance and antibiotic use. Unbiased metagenomic next-generation sequencing (mUNGS) represents a powerful tool to perform such sentinel viral surveillance during the acute phase of illness and could fill, at least partially, some gaps in our understanding of the aetiology of fever [4]. As a by-product, this approach also allows characterization of the virome in children living in this part of the world.

In this study, mUNGS was performed on serum samples collected from hospitalized children (age 0–15 years) admitted with fever (i.e. rectal or axillary temperature ≥38°C), without any exclusion criteria, at the Albert Schweitzer Hospital in Lambaréné, Gabon, between August 2015 to March 2016. A total of 405 samples were collected as part of a previous study [5], of which 385 had a sufficient leftover volume. Of these samples, 360 were randomly selected and were grouped in 18 pools of 20 samples each (6 ul of each of the 20 sera samples per pool were used, corresponding to a volume of 120 ul/pool) according to four age subgroups [6]. We differentiate children below and over 5 years-old (y.o). These 360 sera were aggregated into nine pools for the “neonate/infant” subgroup, four pools each for the below and over 5 y.o subgroups, and one pool for the “adolescent” subgroup (Figure 1(a)). Two “no-template” negative controls were submitted to the entire mUNGS procedure to check for potential contaminants from environmental or experimental sources. Two positive controls (canine distemper virus-spiked samples) were used to assess the mUNGS process efficiency. Each pool/control was treated using a previously published RNA procedure [7], and libraries were prepared using the TruSeq total RNA preparation protocol (Illumina, San Diego, US) with dual indexing. Each library (corresponding to one pool) was loaded individually in a single lane on the HiSeq 4000 platform (Illumina) using the 2 × 100-nucleotide read length protocol. The mean total number of read pairs obtained per pool was 329 038 830 (range 214 348 410–381 090 836, Supplementary Table S1). Reads were analyzed using two methods performed in parallel (Supplementary Figure S1): (1) a bioinformatic pipeline that used virusscan 1.0 (https://github.com/sib-swiss/virusscan) to map reads against the Virosaurus database (version V90v_2018_11) (https://viralzone.expasy.org/8676) which is designed to report any known vertebrate viruses, and (2) by *de novo* assembly. A result was considered positive and reported only if not detected in the “no-template” and positive controls, and if ≥ 300 nucleotides of coverage was obtained. Of note, the use of the RNA protocol does not restrict the mUNGS analysis to the detection of RNA viruses-related sequences and will also detect DNA viruses, particularly in cases of ongoing viral replication (i.e. RNAs are generated). The raw sequence data were deposited in the NCBI Sequence Read Archive under BioProject accession number PRJNA602599.

**Figure 1.**
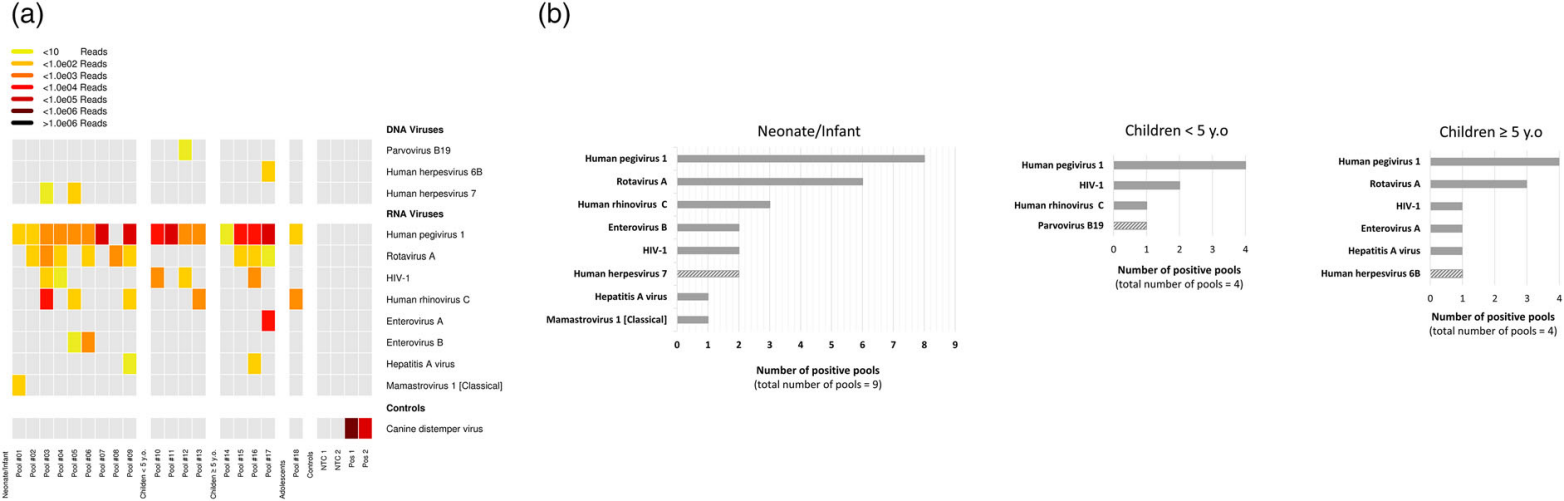
Blood viral sequence analysis by mUNGS. (a) Grid plot of vertebrate virus sequences detected by mUNGS. The approximate number of reads matching the indicated virus genome in each pool is represented by colour code. Pools are grouped in the grid according to the four age-subgroups. Due to limited neonatal patients (*n* = 2), these samples were pooled with infants (both in pool #01). (b) Frequency of sequences detected from viruses of recognized clinical significance in all pools from the same age-subgroup. RNA viruses are represented with filled grey lanes. DNA viruses are represented with dashed grey lanes. The “adolescent” subgroup is not included as it is comprised of only a single pool. HIV-1: *Human immunodeficiency virus 1*, NTC: no-template negative controls, Pos: positive controls.

The mUNGS analysis revealed that at least one virus recognized to cause disease in humans was present in 15/18 (83.3%) pools (Figure 1(a)). Overall, *Rotavirus A* and *Enterovirus* species (including *Enterovirus A/B* and *Human rhinovirus C* (HRV-C)) were the most frequently detected viruses (9/18 (50%) and 8/18 (44.4%) pools, respectively). The detected viruses at the level of each age subgroup, in order of their prevalence, were as follows (Figure 1(b)): In the nine “neonate/infant” pools: *Rotavirus A* (*n* = 6), HRV-C (*n* = 3), *Enterovirus B* (*n* = 2), *Human immunodeficiency virus 1* (HIV-1) (*n* = 2), *Human herpesvirus type 7* (*n* = 2), *Hepatitis A virus* (*n* = 1) and *Mamastrovirus 1* (*n* = 1); in the four “under 5 y.o” pools: HIV-1 (*n* = 2), HRV-C (*n* = 1) and *Parvovirus B19* (*n* = 1); in the four “over 5 y.o” pools: *Rotavirus A* (*n* = 3), HIV-1 (*n* = 1), *Enterovirus A* (*n* = 1), *Hepatitis A virus* (*n* = 1) and *Human herpesvirus type 6B* (*n* = 1); the pool of the “adolescent” subgroup was found to be positive for HRV-C sequences. Our finding that *Rotavirus A* was the most frequently reported virus of recognized clinical significance in both the “neonate/infant” and “over 5 y.o” subgroups confirms previous studies that reported that rotavirus infections frequently generate viremia in the paediatric population [8,9]. The presence of enterovirus species was observed in all age subgroups, with various HRV-C genotypes (argued to be potentially more virulent than HRV-A and -B in children [10]) detected in the “neonate/infant”, “under 5 y.o”, and “adolescent” subgroups. Additionally, *Enterovirus B* was detected in the “neonate/infant” subgroup, and *Enterovirus A* was detected in the “over 5 y.o” subgroup. Among *Enterovirus A* and *B*, the typing analysis revealed the presence of coxsackievirus A5 and echovirus 25 (Supplementary Table S1).

In addition to the viruses recognized to cause diseases in humans listed above, the mUNGS investigations also revealed *Human pegivirus-1* (HPgV-1) sequences in all but one pool (Figure 1(a)). HPgV-1 is known to infect humans but no causal association with disease has been recognized thus far [11].

In conclusion, our mUNGS investigations support evidence that febrile disease of viral origin among children in Sub-Saharan Africa are frequently associated with “common” viruses. The mUNGS data are concordant with the PCR screening results from a previous study [5] that reported the detection of *Human herpesvirus type 6* in the blood of 33% of a subset of 89 patients, *Enterovirus* and *Human rhinovirus* in throat swabs of 1.6% of 191 patients each, as well as *Rotavirus* (11.3% of patients) and *Mamastrovirus 1* (4.8% of patients) in stools. Interestingly in the previous study, *Human herpesvirus type 6* was detected by PCR in patients without exanthema which suggested past infections or acute infections without apparent skin lesions. Of note, the detection of *Human herpesvirus type 6B* (Pool #17) by the mUNGS RNA procedure may suggest an ongoing viral replication. Although mUNGS data should be interpreted with caution, as the detection of viral RNA sequences does not establish that a specific virus is necessarily the cause of patient admission, our results contribute to a better understanding of the potential proportion of viruses that cause fever and thus could help to improve existing clinical algorithms and the subsequent use of antimicrobial agents. Indeed, except for HPgV-1, all RNA and DNA viruses reported by our mUNGS analysis represent commonly recognized viral aetiologies of febrile illness in both paediatric and adult populations across all continents. Among viruses known to cause outbreaks in this part of the world, by mUNGS we did not detect any flavivirus or alphavirus sequences, which confirms the PCR results previously reported [5]. Using an unbiased approach such as mUNGS in specific populations, according also to the epidemiological season, will help to characterize viruses causing fever, and provides a surveillance tool for emerging viral diseases.

## Supplementary Material

Supplemental Material
