# Supplementary material for: Unbiased metagenomic next-generation sequencing of blood from hospitalized febrile children in Gabon
Source: Emerg Microbes Infect. 2020 Jun 11;9(1):1242–4. doi: 10.1080/22221751.2020.1772015 (PMC7448917; doi:10.1080/22221751.2020.1772015)
Supplement: Supplemental Material [file TEMI_A_1772015_SM4404.zip › 177015_Suppl/Table S1.docx]

| **Pool** | **Total number of reads** | **Virus** | **Mapping Virosaurus number of**  **reads (genome coverage (bp))** | ***de novo* assembly number of**  **reads (genome coverage (bp))** |
| --- | --- | --- | --- | --- |
|  |  |  |  |  |
| Pool #01 | 738977406 | *Human pegivirus 1* | 75 (2479) |  |
|  |  | *Mamastrovirus-1* | 13 (368) |  |
|  |  |  |  |  |
| Pool #02 | 629627762 | *Rotavirus A* | 53 (2659) |  |
|  |  | *Human pegivirus 1* | 26 (1596) |  |
|  |  |  |  |  |
| Pool #03 | 762181672 | Human rhinovirus C23 | 1601 (6305) | 25090 (7051) |
|  |  | Human rhinovirus C28 |  | 3401 (6572) |
|  |  | *Rotavirus A* | 234 (7989) |  |
|  |  | *Human pegivirus 1* | 219 (5154) | 1000 (1691) |
|  |  | HIV-1 | 28 (1244) |  |
|  |  | *Human herpesvirus 7* | 9 (451) |  |
|  |  |  |  |  |
| Pool #04 | 728785894 | *Human pegivirus 1* | 451 (5911) | 1197 (1450) |
|  |  | *Rotavirus A* | 25 (1167) |  |
|  |  | HIV-1 | 7 (376) |  |
|  |  |  |  |  |
| Pool #05 | 707321150 | *Human pegivirus 1* | 220 (5918) |  |
|  |  | Human rhinovirus C51* | 87 (3236) | 266 (1078) |
|  |  | *Human herpesvirus 7* | 16 (350) |  |
|  |  | *Enterovirus B* | 5 (304) |  |
|  |  |  |  |  |
| Pool #06 | 724156804 | *Enterovirus B* (E25) | 983 (3372) | 45140 (7358) |
|  |  | *Human pegivirus 1* | 170 (3693) | 1003 (1443) |
|  |  | *Rotavirus A* | 68 (2004) |  |
|  |  |  |  |  |
| Pool #07 | 507713274 | *Human pegivirus 1* | 42221 (8734) | 78208 (5904) |
|  |  |  |  |  |
| Pool #08 | 742547114 | *Rotavirus A* | 185 (7740) |  |
|  |  |  |  |  |
| Pool #09 | 744798292 | *Human pegivirus 1* | 31519 (8364) | 49156 (6823) |
|  |  | *Rotavirus A* | 99 (4165) |  |
|  |  | *Human rhinovirus C* | 27 (666) |  |
|  |  | *Hepatitis A virus* | 5 (368) |  |
|  |  |  |  |  |
| Pool #10 | 457499218 | *Human pegivirus 1* | 2692 (5853) | 37823 (9404) |
|  |  | HIV-1 | 442 (5435) | 9224 (8845) |
|  |  |  |  |  |
| Pool #11 | 525110692 | *Human pegivirus 1* | 11325 (8657) | 604559 (9021) |
|  |  |  |  |  |
| Pool #12 | 748654152 | *Human pegivirus 1* | 941 (6011) | 23215 (9413) |
|  |  | HIV-1 | 25 (551) |  |
|  |  | *Parvovirus B19* | 5 (300) |  |
|  |  |  |  |  |
| Pool #13 | 736362900 | Human rhinovirus C24* | 129 (5458) |  |
|  |  | *Human pegivirus 1* | 104 (3683) | 141 (1106) |
|  |  |  |  |  |
| Pool #14 | 652366342 | *Human pegivirus 1* | 9 (459) |  |
|  |  |  |  |  |
| Pool #15 | 561030276 | *Human pegivirus 1* | 3389 (8064) | 24732 (9266) |
|  |  | *Rotavirus A* | 14 (1050) |  |
|  |  |  |  |  |
| Pool #16 | 746112446 | *Human pegivirus 1* | 2218 (7448) | 15165 (5959) |
|  |  | HIV-1 | 834 (5953) | 18678 (9262) |
|  |  | *Rotavirus A* | 67 (3602) |  |
|  |  | *Hepatitis A virus* | 46 (2210) |  |
|  |  |  |  |  |
| Pool #17 | 703455664 | *Human pegivirus 1* | 20152 (8362) | 63004 (2099) |
|  |  | *Enterovirus A* (CVA5) | 4339 (1173) | 439507 (7388) |
|  |  | *Human herpesvirus 6B* | 15 (921) |  |
|  |  | *Rotavirus A* | 8 (482) |  |
|  |  |  |  |  |
| Pool #18 | 428696820 | Human rhinovirus C24* | 183 (6212) | 52 (1014) |
|  |  | *Human pegivirus 1* | 13 (918) |  |
