# Supplementary figures and images for: Unbiased metagenomic next-generation sequencing of blood from hospitalized febrile children in Gabon
Source: Emerg Microbes Infect. 2020 Jun 11;9(1):1242–4. doi: 10.1080/22221751.2020.1772015 (PMC7448917; doi:10.1080/22221751.2020.1772015)

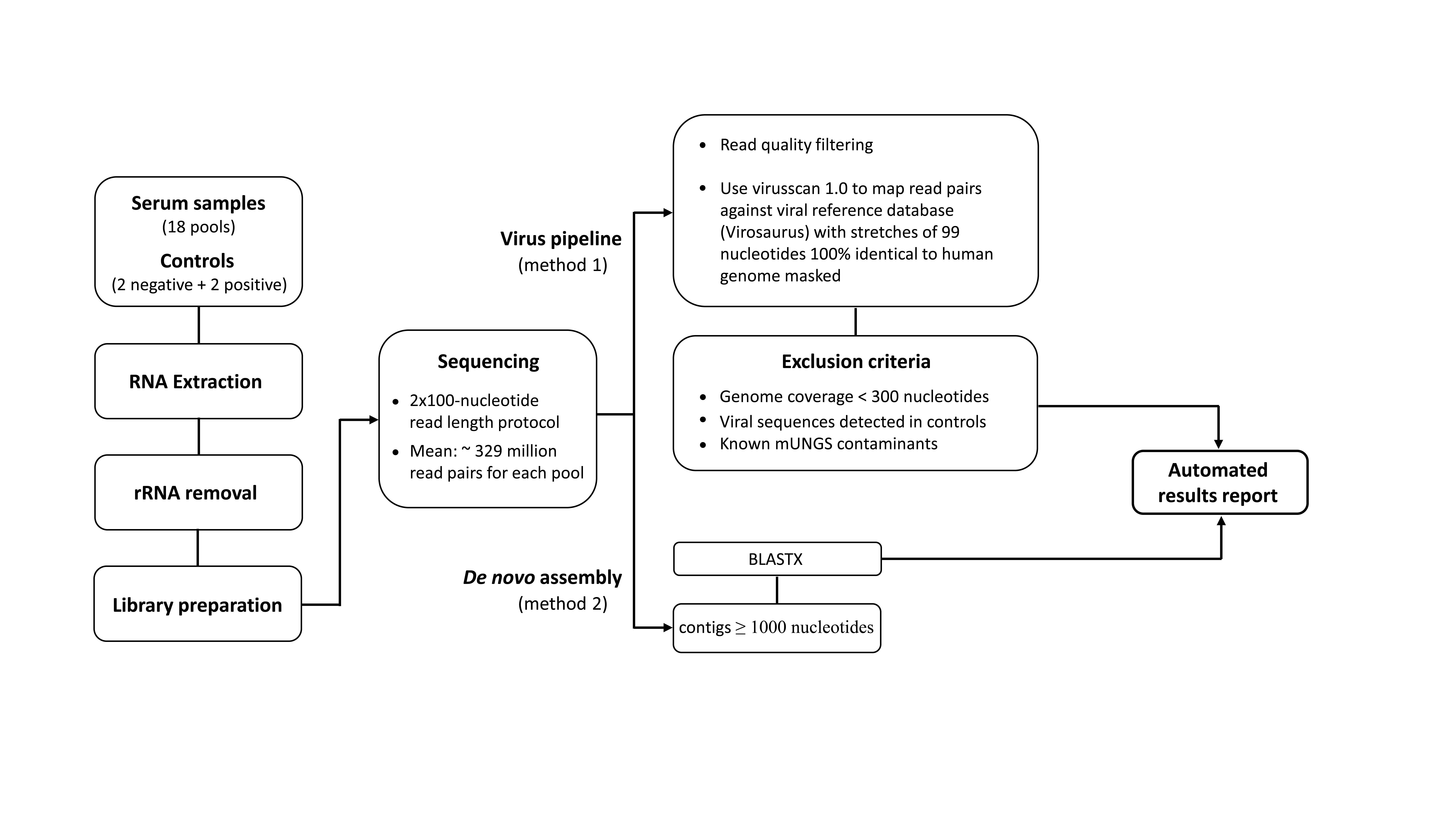

Supplement: Supplemental Material [file TEMI_A_1772015_SM4404.zip › 177015_Suppl/Supplementary Figure S1.jpg]
